# Cross-sectional study on exercise-related skin complaints among sports students at two German universities

**DOI:** 10.1038/s41598-024-62357-9

**Published:** 2024-05-23

**Authors:** Karl Philipp Drewitz, Claudia Hasenpusch, Florian Kreuzpointner, Ansgar Schwirtz, Adolf Klenk, Christian J. Apfelbacher

**Affiliations:** 1 Institute of Social Medicine and Health Systems Research, University of Magdeburg, Leipziger Str. 44, 39210 Magdeburg, Germany; 2 Prevention Center, Faculty for Sport and Health Sciences, Technical University of Munich, 80992 Munich, Germany; 3 Department of Biomechanics in Sports, Faculty for Sport and Health Sciences, Technical University of Munich, 80992 Munich, Germany; 4 Dr. Kurt Wolff GmbH & Co. KG, 33611 Bielefeld, Germany; 5 Family Medicine and Primary Care, Lee Kong Chian School of Medicine, Nanyang Technological University, Singapore, 636921 Singapore

**Keywords:** Exercising, Dermato-epidemiology, Skin complaints, Sports dermatology, Epidemiology, Skin diseases

## Abstract

Sports activities can lead to exercise-related skin complaints. These include different symptoms (e.g. infections, mechanical injuries, contact dermatitis). Previous studies mostly focused only on skin infections and injuries in competitive athletes. The purpose of this study was to determine the frequency and characteristics of exercise-related skin complaints among sports students and to what extent these complaints influence physical fitness. We performed a self-administered online survey among 259 actively exercising sports students from two German universities. Descriptive analyses were conducted. The most common complaints were blistering (57.3%), dryness (56.7%), redness (44.7%), and chafing (34.0%). Hands and feet (78.0% each) were most frequently affected. Participants whose skin was particularly stressed (47.5%) had higher training duration (7.6 h/week, 95%-CI 6.8–8.3 h) than those without complaints (5.1 h/week, 95%-CI 5.5–6.7 h, p = 0.003). The students reported reduced intensity (34.7%) and frequency (22.7%) of training due to their skin complaints. A reduction in performance was reported by 32.0% of the students. Actively exercising sports students considered an intact skin as essential for their physical fitness. Reported impairments of the skin led to a reduced intensity and frequency of training. To enhance the awareness of exercise-related skin complaints, further research is necessary.

## Introduction

Increased sports activities can lead to exercise-related skin complaints comprising a variety of different skin diseases and symptoms^1–3^. Inflammatory skin conditions (e.g. contact dermatitis) or skin infections are most common in exercising, especially in actively exercising students or athletes^2^. Infectious diseases of the skin are caused by exposure to fungi (e.g. tinea), viruses (e.g. herpes simplex virus, human papilloma virus), bacteria (e.g. impetigo contagiosum) or parasites^4^. Furthermore, the skin is also exposed to various strains and stresses such as environmental factors (e.g. UV radiation, high and low temperatures), allergens^5,6^ and to mechanical stress^2^. Chronic pressure, close body contact or long-term and repetitive friction on the same location on the skin can cause, for example, hyperkeratoses and calluses^4,7^. Summarised as ‘frictional dermatoses’ these precursors of a serious skin disease are so far under-recognised apart from a few articles from the field of occupational dermatology^8^.

Most of the existing studies on exercise-related skin diseases focus on infectious skin disorders^2,9–14^ or only on competitive (high school) athletes in relation to a specific discipline such as wrestling, swimming, baseball, running or ice-hockey^15–19^.

Exercise-related skin complaints can develop long before a disease manifests itself^2,5^. From a prevention perspective, it therefore seems appropriate to recognise these precursors at an early stage, to sensitise actively exercising sports students to them and to open up simple ways of counteracting the symptoms or complaints. As actively exercising sports students also usually practise intensively and often take part in competitions, physical fitness is essential. Physical fitness can particularly be impaired by skin complaints. They can even thwart sports students from doing their classes or from participating in competitions^5^.

The aim of this present study was to determine the frequency and characteristics of exercise-related skin complaints among actively exercising sports students in Germany and to what extent these complaints influence training or competitiveness.

## Participants and methods

### Survey development

We developed a questionnaire based on a previous literature research and expert discussions to identify essential topic-related items for creating an item pool. To ensure face validity three experts reviewed the questionnaire draft items of relevance and comprehension. Afterwards, we tested the questionnaire among medical and sports students. On basis of the results, designed items were revised or removed according to the recommendations of the experts. The questionnaire included 35 items in four domains: (a) frequency and type of skin complaints associated with exercising and degree of influence on performance (n = 15), (b) individual approaches to the occurrence of skin complaints and effects on skin condition (n = 7), and (c) general skin condition, skin care and physician-diagnosed skin diseases (n = 5) as well as (d) sociodemographic items (n = 8). The questionnaire design included closed, open and semi-open questions (Supplementary Information).

### Study design and data collection

We conducted a self-administered online survey through Unipark by Tivian (formerly Questback). It was administered among sports students at two German universities: Technical University Munich (TUM) and Otto von Guericke University (OVGU) Magdeburg. The survey was advertised with flyers and posters at TUM, which contained a QR code to call up the questionnaire directly. At OVGU Magdeburg, the survey was advertised directly via the lecturers in the sports science department. Participation was incentivised by a raffle.

The data collection lasted from April 1 to June 30, 2020 at the TU Munich and from June 17 to July 31, 2020 at the OVGU Magdeburg.

### Data management and analysis

In the context of the online survey, the collected data were analysed descriptively using SPSS version 26. Absolute and relative frequencies were evaluated. Differences between mean values of different groups were compared using the Student’s *t*-test. To assess differences of categorical variables between groups, the chi-square independence test was used. A p-value less than 0.05 was considered statistically significant. Incomplete questionnaires were included in the statistical analysis. Taking into account the missing values, the presentation of the relative frequencies refers to the totality of the questions answered by the students in each case. The free text answers were structured according to topic content and summarised in inductively formed categories.

### Ethical approval and consent

The Ethics Committee of the University of Regensburg (18-970-101) approved the study protocol and all necessary procedures. All participants gave informed consent prior to the survey. Data collection for the survey was anonymous. The online consent form of the participants also did not require any personal information. Only participants, who had previously read and confirmed the consent form, could answer the questionnaire. All research was performed in accordance with relevant guidelines and regulations, e.g. the Declaration of Helsinki^20^.

## Results

### Characteristics of study population

In the survey, 259 students participated (229 at TUM, 30 at OVGU). The majority (n = 90) was 20–21 years old (range 17–32 years). Most participants (n = 200) were enrolled in a bachelor’s program and studied in the first (n = 66) or second (n = 48) semester (range 1st-13th semester), 45.7% were female, almost half (n = 124) stated that they also take part in competitions. On average, all participants exercised 6.8 ± 3.9 h per week (range 0.5–21 h). The demographic characteristics of the study population are summarised in Table 1.

**Table 1:** Sociodemographic characteristics of the study population.

	Total study population	Sub study population	
	(n = 259)^a^	TU Munich (n = 229)^a^	OVGU Magdeburg (n = 30)
Gender, n (%)^a^			
Female	107 (45.7)	90 (44.1)	17 (56.7)
Male	127 (54.3)	114 (55.9)	13 (43.3)
Total	234^a^ (100.0)	204^a^ (87.1)	30 (12.9)
Mean age (range), years	21.45 (17–32)	21.53 (17–32)	21.44 (17–31)

^a^25 missing values on gender.

### Types of sport

**Figure 1:**
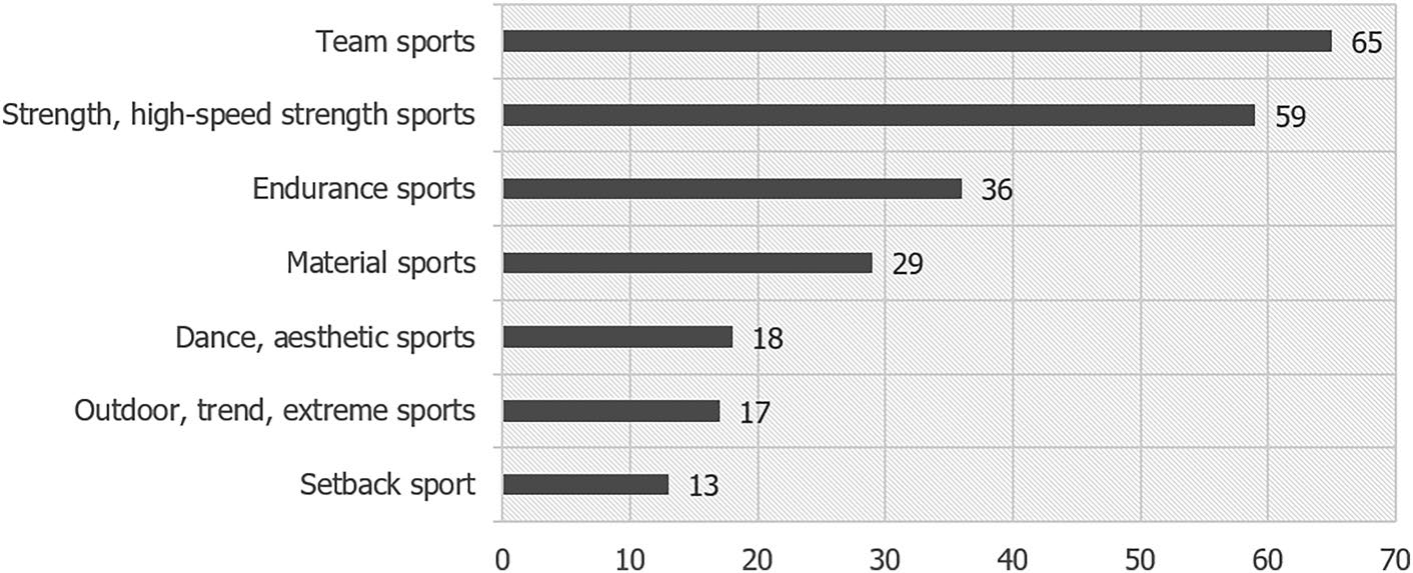
Participants’ main sport types (n).

Participants reported 60 different main sport types. The most common types included team sports (27.9%), strength and high-speed sports (25.3%) and endurance sports (15.5%). Among the students, who practiced team sports, the majority played soccer (13.3%) and volleyball (5.6%). Within the group of strength and high-speed sports, fitness (10.7%) and weight training (8.5%) were indicated most often. Running (6.9%) and swimming (3.9%) were most common endurance sports. Main types of sport are presented in Fig. 1.

### Skin complaints in connection with exercising

Almost half of the participants (n = 123, 47.5%) stated that their skin was particularly stressed by exercising. Male and female participants suffered similarly from a stressed skin condition (50.9% vs. 49.1%). In 80 of the participating students (30.9%), the skin reacted more sensitively due to exercising.

The most common skin complaints among the participants were blistering (57.3%), dryness (56.7%), redness (44.7%), and chafing (34%). The extremities were most frequently affected, especially hands and feet (78% each).

The participants whose skin was particularly stressed by the training statistically significantly exercised more hours per week than those without skin complaints (7.6 h/week, 95%-CI 6.8–8.3 vs. 5.1 h/week, 95%-CI 5.5–6.7, p = 0.003). For the statement that the skin reacted more sensitively, no statistically significant differences were found in relation to the training duration: 7.5 h/week (6.5–8.5 h) vs. 6.5 h/week (6.0–7.0 h).

### General skin complaints

The majority (n = 136, 52.5%) reported suffering from skin complaints permanently or temporarily regardless exercising (Table 2).

**Table 2:** Non-exercise-related skin complaints.

	n	Valid percent
Permanently	29	11.2
Temporarily	107	41.3
No skin complaints	123	47.5
Total	259	100.0

**Figure 2:**
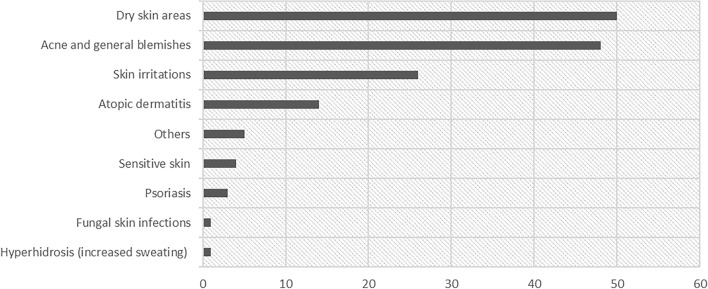
General skin complaints (n),*others included such as oily skin, pustules, eczema.

The general skin complaints included dry skin areas (n = 50), acne and general skin blemishes (n = 48), atopic dermatitis (n = 14), and skin irritations which comprised redness, itching, or allergic reactions (n = 26), see Fig. 2.

In addition, 34 participants (13.1%) reported existing skin problems since childhood, 34.0% (n = 88) since adolescence and 6.6% (n = 17) since adulthood.

### Skin diseases (self-reported diagnoses)

**Figure 3:**
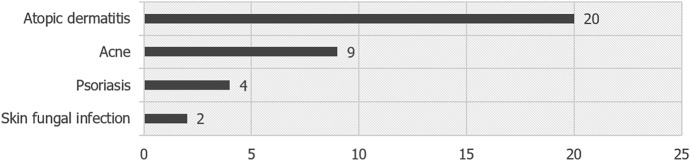
Self-reported skin diseases (n).

Physician-diagnosed skin diseases were reported by 16.7% participants (male n = 21, female n = 18), such as atopic dermatitis (n = 20) and acne (n = 9), see Fig. 3. There were two participants with a physician-diagnosed fungal infection.

### Impact on physical fitness

36.0% and 14% of the participants indicated that an intact skin was “important” or “very important” for their physical fitness. For 60 participants (40.0%), intact skin played a less important role with regard to their physical fitness. Only 10% of the participants reported being “dissatisfied” with their performance in training due to skin complaints. In contrast, 54.0% (n = 81) stated that they adopted a protective posture due to skin complaints. In connection with the reported complaints, the participants indicated that they reduced the intensity (34.7%, n = 52) and frequency (22.7%, n = 34) of their training as a result. 28 participants (32.0%) reported a reduction in their physical fitness.

### Individual approaches to dealing with skin complaints

Two third of the participants (n = 102, 68%) reported that they had already taken measures to combat their skin complaints. Table 3 shows different measures in dealing with the skin complaints. In contrast, the majority of the participants (79.4%) have not yet tried to use prescription drugs or over the counter pharmaceutical products (53.9%) to treat their skin complaints, nor did they change their care products (58.8%) or sportswear (58.8%).

**Table 3:** Measures taken against the skin complaints.

Measures taken against the skin complaints	Improved		Unchanged		Declined		Not attempted	
	n	%	n	%	n	%	n	%
Application of a cosmetic product (drugstore product)	43	42.2	18	17.6	2	2.0	39	38.2
Application of a pharmaceutical skin care product	36	35.3	9	8.8	2	2.0	55	53.9
Use of a prescription drug	16	15.7	5	4.9	–	–	81	79.4
Use of bandages, tapes etc	42	41.2	37	36.3	4	3.9	19	18.6
Change of a previous care product	14	13.7	10	9.8	1	1.0	77	75.5
Change of sportswear	30	29.4	11	10.8	1	1.0	60	58.8
Change of the sports equipment	10	9.8	80	78.4	–	–	12	11.8
Other^a^	3	2.9	–	–	–	–	46	45.1

– No data.

^**a**^Other measures included application of hand cream, change of posture during sports activity, frequent washing of sportswear.

In connection with exercising, 17.1% of the participants used medical care products. 63.7% of the participants (n = 65) used bandages or tapes to alleviate skin complaints. This measure showed a noticeable improvement in the skin appearance for 41.2% (n = 42). The use of cosmetic products was taken as a measure by more than half of the participants (61.8%, n = 63) and led to an improvement of the complaints in 42.2% (n = 43) of them (Table 2).

In 46.1% (n = 47) of those who applied a pharmaceutical skin care product, 76.6% (n = 36) showed an improvement in skin symptoms. In addition, 41.2% (n = 42) changed their sportswear to alleviate the skin complaints. This procedure had a successful effect in 71.4% (n = 30). A change of the previous care product was undertaken by 24.5% (n = 25), which showed a successful improvement in 56.0% (n = 14). Another measure to achieve an improvement in skin complaints was a change in sports equipment for 21.6% (n = 22), of which 45.5% (n = 10) also achieved a positive effect on the skin condition. Only 20.6% (n = 21) took a drug prescribed by a physician for their skin complaints, which resulted in relief for 76.2% (n = 16).

49% (n = 50) of the participants who noticed an alleviation of skin complaints using cosmetic products, pharmaceutical products and other measures reported also an improvement in their physical fitness.

## Discussion

To the best of our knowledge, we present the first study on self-reported exercise-related skin complaints in actively exercising sports students in Germany. Almost half of the participants (47.5%) reported that their sporting activities stressed their skin, whereby an intact skin was considered essential for physical performance. Duration of exercising was related to the skin complaints. The majority of study participants did not consider to treat their skin complaints with pharmaceutical products nor to change every day care products or sportswear. Possible explanations may be that skin complaints have diminished after a short time or the uncomfortable feeling to see a physician. Further, it can be assumed that participants have not been aware that those skin complaints are directly exercise-related.

Sports-related skin diseases are more difficult to recognise as such by the affected individuals and diagnosis and treatment can also be more challenging^9^. The treatment of skin diseases, such as skin infections, in actively exercising individuals can differ compared to the disease management of the general population due to their demands on their own functionality and avoidance of loss of training time^9^. With regard to managing skin problems, our study participants initially tended to use bandages and tapes to protect the skin as well as cosmetic products to alleviate their skin complaints compared to medications prescribed by a physician. The results indicated that the measures led to an improvement in the exercise-related skin complaints. However, the majority of participants stated that they did not attempt or took any approaches against the complaints associated with exercising. Although 40% stated that an intact skin is a significant factor in physical fitness and training performance, only 10% of the respondents stated that they were dissatisfied with their training performance due to skin complaints.

Skin complaints among our participants varied widely but were mostly localised at the extremities. According to Carr and Cropley, infectious skin diseases and mechanically induced skin diseases are most common exercise-related diseases^2^. Our data showed, that diagnosed dermatoses such as infectious skin disease were relatively small (n = 2) and only 24 students were diagnosed with an inflammatory skin disease (atopic dermatitis, n = 20; psoriasis, n = 4). However, it remains unclear if these self-reported medical diagnoses were directly associated with exercising.

Our study comprised sports students at two German Universities and we were able to cover and evaluate multiple sports disciplines (e.g. team and individual sports). Compared to other cross-sectional studies, not only one skin disease was evaluated but skin complaints in general.

Despite intensive recruitment and incentivisation by a raffle, a 10% participation rate was achieved at the Munich (TUM) location and a 30% participation rate at the Magdeburg (OVGU) location. The unbalanced number of participants (TUM vs. OVGU) might be closely related to the COVID-19 pandemic and the resulting restrictions in university and study operations; the sample represents only a fraction of the total number of the target population, which reduces the generalizability of the findings. The high number of missing values, e.g. for gender (approximately 10%), makes the evaluation and transferability of the results difficult. During the study, we identified improvements to the questionnaire for possible follow-up research. For example, the questionnaire on measures to combat skin complaints did not take into account which of those affected sought medical help because of the skin complaints. Also, the use and possible modification of sportswear and training equipment should be investigated in greater depth as well as the type and frequency of cleaning of these items.

Literature on exercise-related skin complaints is scarce. Regarding skin diseases in actively exercising people, studies mostly focus on skin infections^9,12,21,22^. We only identified one comparable study from Liebich et al.^23^ They investigated self-reported exercise-related dermatoses in 492 actively exercising high school and university students as well as athletes from squads (aged 14–35, 46% female, 53% elite athletes, data collection between 2013 and 2019). They compared recreational (exercising less than 8 h per week) and elite athletes (exercising eight or more hours per week) in lights of frequency of dermatoses. In their sample, athletes exercising 9–15 h/week reported fewer skin diseases than participants, who exercised only 2–5 h/week. Further, female participants reported more skin diseases than male participants did. We did not have such findings in our data. Further, we did not find any publications on surveys about how actively exercising students deal with skin complaints related to exercising.

## Conclusion

Skin complaints related to exercising seem to be a burden on actively exercising sports students. An intact skin is considered essential for their physical fitness. However, most of them do not seek medical consultation or use pharmaceutical products to alleviate those complaints. Further research seems necessary to identify and distinguish exercise-related complaints and actual skin diseases and how they impact training, competitiveness and health-related quality of life.

### Supplementary Information

Supplementary Information.

## Data Availability

Data can be obtained from the corresponding author upon reasonable request.

## References

[1] Adams BB (2006). Sports Dermatology.

[2] Carr PC, Cropley TG (2019). Sports dermatology: Skin disease in athletes. Clin. Sports Med..

[3] Pecci M, Comeau D, Chawla V (2009). Skin conditions in the athlete. Am. J. Sports Med..

[4] Adams BB (2002). Dermatologic disorders of the athlete. Sports Med..

[5] Moehrle M, Blum A (2004). Haut und sport: Skin and sports. J. Dtsch. Dermatol. Ges..

[6] Liebich C, Wergin VV, Schubert I, Von Bruehl ML, Marquart C, Halle M, Oberhoffer R, Wolfarth B (2021). Dermatoses in competitive athletes. Dtsch. Z. Sportmed..

[7] Bennike NH, Andersen KE, John SM, Duus Johansen J, Rustemeyer T (2020). Frictional trauma/mechanic skin diseases. Kanerva’s Occupational Dermatology.

[8] Arora G, Khandpur S, Bansal A (2023). Current understanding of frictional dermatoses: A review. Indian J. Dermatol. Venereol. Leprol..

[9] Nowicka D, Bagłaj-Oleszczuk M, Maj J (2020). Infectious diseases of the skin in contact sports. Adv. Clin. Exp. Med..

[10] Ashack KA, Burton KA, Johnson TR (2016). Skin infections among US high school athletes: A national survey. J. Am. Acad. Dermatol..

[11] Minooee A, Wang J, Gupta GK (2015). Sports: The infectious hazards. Microbiol. Spectr..

[12] Peterson AR, Nash E, Anderson BJ (2019). Infectious disease in contact sports. Sports Health.

[13] Adams BB (2008). Skin infections in athletes. Dermatol. Nurs..

[14] Weesner T (2017). Skin infections: Which student-athletes are at greatest risk?. NASN Sch. Nurse.

[15] Farhadian JA, Tlougan BE, Adams BB (2013). Skin conditions of baseball, cricket, and softball players. Sports Med..

[16] Mailler-Savage EA, Adams BB (2006). Skin manifestations of running. J. Am. Acad. Dermatol..

[17] Gordy MA, Cobb TP, Hanington PC (2018). Swimmer’s itch in Canada: A look at the past and a survey of the present to plan for the future. Environ. Health.

[18] Herzog MM, Fraser MA, Register-Mihalik JK (2017). Epidemiology of skin infections in men’s wrestling: Analysis of 2009–2010 through 2013–2014 National Collegiate Athletic Association surveillance data. J. Athl. Train..

[19] Tlougan BE, Mancini AJ, Mandell JA (2011). Skin conditions in figure skaters, ice-hockey players and speed skaters: Part II—Cold-induced, infectious and inflammatory dermatoses. Sports Med..

[20] World Medical Association (2013). Declaration of Helsinki: Ethical principles for medical research involving human subjects. Jama.

[21] Grosset-Janin A, Nicolas X, Saraux A (2012). Sport and infectious risk: A systematic review of the literature over 20 years. Med. Mal. Infect..

[22] Paradise SL, Hu Y-WE (2021). Infectious dermatoses in sport: A review of diagnosis, management, and return-to-play recommendations. Curr. Sports Med. Rep..

[23] Liebich C, Wegin VV, Marquart C (2021). Skin diseases in elite athletes. Int. J. Sports Med..

