# Supplementary material for: Cross-sectional study on exercise-related skin complaints among sports students at two German universities
Source: Sci Rep. 2024 May 23;14:11829. doi: 10.1038/s41598-024-62357-9 (PMC11116371; doi:10.1038/s41598-024-62357-9)
Supplement: Supplementary file 1 — Supplementary Information. [file 41598_2024_62357_MOESM1_ESM.pdf]

Universität Regensburg

«ID»

Questionnaire on sports-associated skin complaints (sports dermatoses)

The skin is the body's largest organ and forms its outer protective covering. It is sometimes subjected to heavy strain during sport. Although the epidermis renews itself about once a month, there is a risk of wear and tear if it is chronically overstressed locally.

This questionnaire serves to record the skin care needs of students who are active in sport. The survey is part of a scientific research project that aims to develop solutions for sports-related skin complaints. The study is being conducted by Prof. Apfelbacher (Medical Sociology, University of Regensburg) in collaboration with Dr. Kreuzpointner (TU Munich). Your data and information will be treated confidentially and will only be scientifically evaluated in anonymized form.

The questionnaire is divided into three parts:
In part A, we would like to know to what extent sports-associated skin complaints restrict you in training or in competition situations.
Part B is about your approach to sports-related skin complaints.
Part C relates to your general skin situation and skin care.

There are different types of questions in this questionnaire. Please mark the answer that applies to you for closed questions and enter free text for open questions.

Please answer the questionnaire as completely as possible!

Screening questions

Do you exercise at least 3 times a week?
Do you do at least 6 hours of sport per week?
What type of sport do you mainly do during this time?

Part A. Restrictions due to sports-related skin complaints

1. Do you have the impression that your skin is particularly stressed by your sporting activities? (manifested e.g. in blisters, redness, dryness, rashes etc.)
   - ○ Yes ○ No
2. Do you have the impression that your skin reacts more sensitively to your sporting activities?
   - ○ Yes ○ No

If you answered "No" to both question 1 and question 2, please skip the questions in parts A and B and continue with part C.

3. 3. what sports-related skin complaints have you had in the last 12 months? This refers to all skin problems that occurred during or in connection with sport, including blisters, calluses, wounds, etc. (multiple choice possible).
   - ○ Dryness
   - ○ Redness
   - ○ Itching
   - ○ Pain
   - ○ Burning
   - ○ Hypersensitivity to previously well-tolerated care products
   - ○ Rash
   - ○ Dandruff on the head
   - ○ Dandruff on the body

- ○ Cracking
- ○ Blistering
- ○ Wheals
- ○ Softened skin
- ○ Callus formation
- ○ Chafing
- ○ Fungal diseases
- ○ Bacterial skin infections
- ○ Hair loss on the body (e.g. due to rubbing of sports clothing or equipment))
- ○ …………………………………………………

4. Which parts of the skin are affected and to what extent?

	Strong	Medium	Low	Not at all
Hands/palms				
Arms/shoulders				
Feet/soles				
Legs/hips				
Head/neck				
Chest/nipples				
Abdomen				
Back				
Buttocks/intimate area				

5. Do the skin complaints affect your training frequency?
   ○ Yes, very much ○ Yes, a little ○ No

6. Do the skin complaints affect your training intensity?
   ○ Yes, very much ○ Yes, a little ○ No

7. Do you have the impression that your performance during training is limited by the skin complaints?
   ○ Yes, very much ○ Yes, a little ○ No

8. Are you dissatisfied with your training performance because of the skin complaints?
   ○ Yes ○ No

9. How often do you take relieving postures because of the skin complaints?
   ○ Very often ○ Often ○ Soemtimes ○ Never

10. Do you prepare your skin for competitions in a special way?
   ○ Yes ○ No ○ I do not participate in competitions

If yes, how do you prepare it? (free text)

11. Do the skin complaints have a negative impact on your performance in competitions?
   ○ Yes ○ No ○ I do not participate in competitions

12. How important is an intact skin for your athletic performance?
   ○ Very important ○ Important ○ Less important ○ Not important

**Part B. Your approach to sports-related skin complaints**
13. Have you already done anything about your skin complaints? (e.g. use of a skin care product, change of sportswear, tapes, etc.)<br>○ Yes ○ No
If you answered "No" to question 13, please skip the remaining questions in Part B and continue with Part C.
14. How have you treated the skin complaints so far and have they improved as a result? (Please tick the appropriate box)

	Improved	Remained the same	Deteriorat ed	Not attempted
Application of a beauty product from the drugstore				
Application of an over the counter pharmaceutical product				
Application of a drug after medical advice				
Application of bandages, tapes, etc.				
Change of a currently used care product				
Change of sportswear				
Change of sports equipment				
Other, namely:				

15. If you use cosmetic products or medical skin care, which products do you use for which skin areas? (Free text in table form)
Have your skin complaints improved as a result? (Please tick)

Product name/ description	Skin area	Strongly improved	Somewhat improved	Remained the same	Deteriorate d

16. Has the treatment of your skin complaints improved your overall athletic performance?
○ Yes, very ○ Yes, somewhat ○ No

17. Were there any negative effects when using a skincare product in sport?
○ Yes ○ No

	If yes, which one? (free text)

18. Do you feel uncomfortable applying skincare products in front of others?
   - o Yes
   - o No
   - o Depends on product

Part C. General skin condition and skin care

19. Do you have any issues with your skin in general (not sports related)?
   - o Yes
   - o No

   If yes, how often do the complaints occur?
   - o Occasionally
   - o Permanently

   If yes, how long have the complaints been present?
   - o Childhood
   - o Adolescence
   - o Adulthood

20. Has a doctor ever diagnosed you with a skin disease?
   - o Yes
   - o No

   If yes, which one? (free text)

21. Which cleaning and skin care products do you generally use in connection with your sporting activity? (free text)

22. Sex: o Female o Male

23. Age:

24. Aimed degree o Bachelor o Master o State exam

25. Study programme

26. Semester:

27. Main Sport:

28. What other sports do you play?

29. How many hours of sport do you do on average per week?

30. Have you taken part in any competitions in the last 24 months?
   - o Yes
   - o No

31. What sporting successes have you had in the last 24 months? (e.g. ran a marathon, won a medal at a competition, etc.)

Thank you very much for answering the questionnaire!
You have been a great help to us.
